# 1-[4-(4-Chloro­phen­yl)piperazin-1-yl]-3-(6-oxo-3,4-diphenyl-1,6-dihydro­pyridazin-1-yl)propan-1-one

**DOI:** 10.1107/S160053681203543X

**Published:** 2012-08-15

**Authors:** Abdullah Aydın, Mehmet Akkurt, Deniz S. Doğruer, Orhan Büyükgüngör

**Affiliations:** a Department of Science Education, Faculty of Education, Kastamonu University, 37200 Kastamonu, Turkey; b Department of Physics, Faculty of Sciences, Erciyes University, 38039 Kayseri, Turkey; c Department of Pharmaceutical Chemistry, Faculty of Pharmacy, Gazi University, 06330 Ankara, Turkey; d Department of Physics, Faculty of Arts and Sciences, Ondokuz Mayıs University, 55139 Samsun, Turkey

## Abstract

In the title compound, C_29_H_27_ClN_4_O_2_, the six-membered ring of the pyridazine group is nearly planar [maximum deviation = −0.062 (2) Å] and its mean plane makes dihedral angles of 43.05 (9), 44.71 (10) and 72.57 (9)°, respectively, with the two phenyl and benzene rings. The piperazine ring has a chair conformation and its mean plane is almost perpendicular to the attached benzene ring, with a dihedral angle of 83.20 (16)°. In the crystal, mol­ecules are linked *via* two pairs of C—H⋯O inter­actions, which result in the formation of chains propagating along [10-1]. Neighbouring chains are linked *via* C—H⋯π inter­actions.

## Related literature

For the synthesis and biological activity of the title compound, see; Doğruer *et al.* (2007 ▶). For related structures, see: Aydın *et al.* (2008 ▶); Girisha *et al.* (2008 ▶). For puckering parameters, see: Cremer & Pople (1975 ▶). For standard bond lengths, see: Allen *et al.* (1987 ▶).
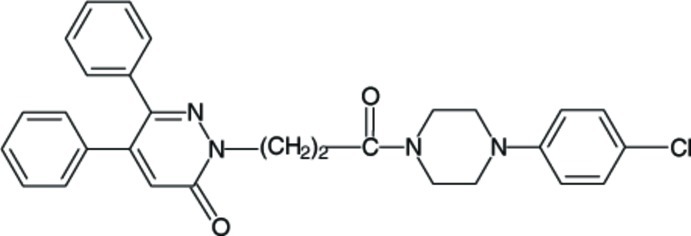


## Experimental

### 

#### Crystal data

C_29_H_27_ClN_4_O_2_
*M* *_r_* = 499.00
Triclinic, 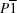

*a* = 10.7929 (10) Å
*b* = 10.8527 (10) Å
*c* = 12.7815 (13) Å
α = 97.745 (8)°
β = 104.041 (7)°
γ = 115.635 (7)°
*V* = 1259.3 (2) Å^3^
*Z* = 2
Mo *K*α radiation
μ = 0.19 mm^−1^
*T* = 296 K
0.66 × 0.53 × 0.35 mm

#### Data collection

Stoe IPDS 2 diffractometer
Absorption correction: integration (*X-RED32*; Stoe & Cie, 2002 ▶) *T*_min_ = 0.784, *T*_max_ = 0.948
15922 measured reflections
4945 independent reflections
3837 reflections with *I* > 2σ(*I*)
*R*_int_ = 0.059

#### Refinement

*R*[*F* ^2^ > 2σ(*F* ^2^)] = 0.041
*wR*(*F* ^2^) = 0.111
*S* = 1.05
4945 reflections
326 parameters
H-atom parameters constrained
Δρ_max_ = 0.29 e Å^−3^
Δρ_min_ = −0.37 e Å^−3^

### 

Data collection: *X-AREA* (Stoe & Cie, 2002 ▶); cell refinement: *X-AREA*; data reduction: *X-RED32* (Stoe & Cie, 2002 ▶); program(s) used to solve structure: *SIR97* (Altomare *et al.*, 1999 ▶); program(s) used to refine structure: *SHELXL97* (Sheldrick, 2008 ▶); molecular graphics: *ORTEP-3 for Windows* (Farrugia, 1997 ▶); software used to prepare material for publication: *WinGX* (Farrugia, 1999 ▶).

## Supplementary Material

Crystal structure: contains datablock(s) global, I. DOI: 10.1107/S160053681203543X/su2489sup1.cif

Structure factors: contains datablock(s) I. DOI: 10.1107/S160053681203543X/su2489Isup2.hkl

Supplementary material file. DOI: 10.1107/S160053681203543X/su2489Isup3.cml

Additional supplementary materials:  crystallographic information; 3D view; checkCIF report

## Figures and Tables

**Table 1:** Hydrogen-bond geometry (Å, °) *Cg*1 and *Cg*4 are the centroids of the N1/N2/C1–C4 and C11–C16 rings, respectively.

*D*—H⋯*A*	*D*—H	H⋯*A*	*D*⋯*A*	*D*—H⋯*A*
C12—H12⋯O1^i^	0.93	2.44	3.323 (2)	158
C25—H25⋯O2^ii^	0.93	2.53	3.289 (3)	139
C10—H10⋯*Cg*1^iii^	0.93	2.88	3.431 (2)	119
C29—H29⋯*Cg*4^iv^	0.93	2.86	3.762 (2)	165

Symmetry codes: (i) 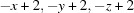; (ii) 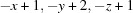; (iii) 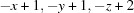; (iv) 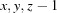.

**Fig. 1.**
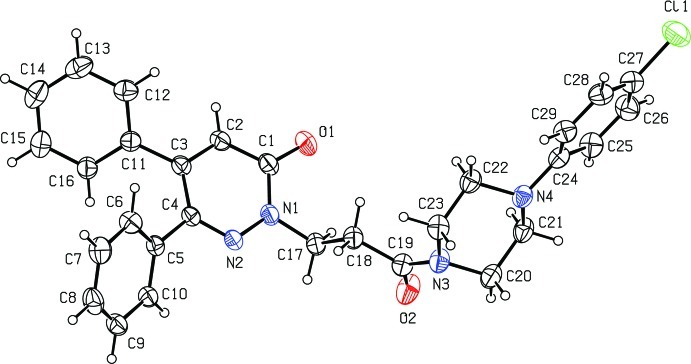
The molecular structure of the title molecule, with the atom numbering. Displacement ellipsoids are drawn at the 30% probability level.

**Fig. 2.**
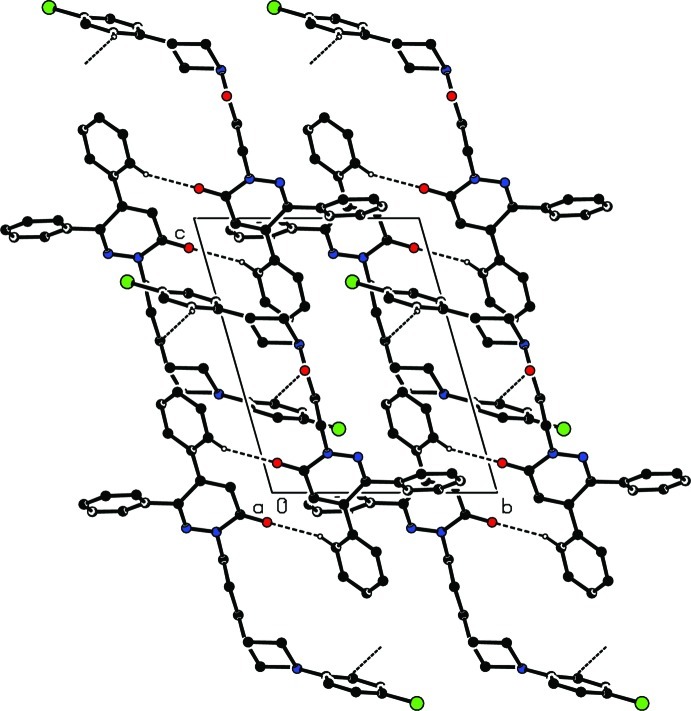
A view along the a axis of the crystal packing of the title compound. The C-H···O interactions are shown as dashed lines [see Table 1 for details; H atoms not participating in these interactions have been omitted for clarity].

## supplementary crystallographic information

### Comment

1-[4-(4-Chlorophenyl)piperazin-1-yl]-3-(6-oxo-3,4-diphenyl-1,6-dihydropyridazin-1-yl)propan-1-one has analgesic and anti-inflammatory effect. Its *in vivo* analgesic and anti-inflammatory activities were tested in mice. This compound showed higher analgesic activity than aspirin at 100 mg/kg. Analgesic activity results of the compound also shows good correlation with its anti-inflammatory activity and produced anti-inflammatory activity in both phases of carrageenan-induced edema (Doğruer *et al.*, 2007).

In the present study, the title compound has been synthesized for first time by (Doğruer *et al.*, 2007) and characterized by spectroscopic techniques. Herein we report on the synthesis and its crystal structure.

In the title compound, Fig. 1, the six-membered ring of the pyridazin-3(2*H*)-one system is nearly planar with maximum deviations of -0.062 (2) Å for N1, 0.052 (2) Å for C1 and -0.045 (2) Å for C3 from the mean plane. The dihedral angles between the mean plane of the six-membered ring (N1/N2/C1–C4) and the phenyl rings C11—C16 and C5—C10 are 44.71 (10)° and 43.05 (9)°, respectively.

As seen in Fig. 1, the Cl1—C27—C28—C29 and N3—C20—C21—N4 torsion angles are 178.69 (14) and 57.22 (18)°, respectively. The double-bond length for C19—O2 is 1.2192 (19) Å and the C27—Cl1 bond length is 1.7450 (19) Å. All bond lengths (Allen *et al.*, 1987) and angles are within normal ranges and are comparable to those reported for similar structures (Aydın *et al.*, 2008; Girisha *et al.*, 2008).

The piperazine ring (N3/N4/C20–C23) has a chair conformation with puckering parameters: Q_T_ = 0.566 (2) Å, θ = 0.5 (2) ° and φ = 67 (10) ° (Cremer & Pople, 1975). The mean plane of the six-membered ring forms a dihedral angle of 83.20 (16)° with the benzene ring (C24–C29). The phenyl rings (C11–C16) and (C5—C10) make dihedral angles of 67.84 (10)° and 32.76 (9)° with the benzene ring (C24–C29), respectively, whilst the dihedral angle between them is 60.42 (10)°.

In the crystal, molecules are linked via two pairs of C—H···O interactions which result in the formation of chains propagating along [1 0 -1], (Table 1 and Fig. 2). Neighbouring chains are linked via C—H···π interactions (Table 1).

### Experimental

The title compound was synthesized according to the literature procedure (Doğruer *et al.*, 2007). 0.01 Mol of compound 3-[5,6-Diphenyl-3(2*H*)-pyridazinone-2-yl] propanoic acid in 40 ml dichloromethane at 273 K (ice-bath) was treated with triethylamine (1 ml) and 0.01 mol of ethyl chloroformate. After stirring the reaction mixture at 273 K for 15 min, 0.011 mole of 4-chlorophenylpiperazine was added to this solution. This mixture was stirred at 273–298 K for 24 h and evaporated to dryness. The product was then solidified with ice-cold water and recrystallized from ethanol (yield 52%, M.p. 432 K). IR *v* cm^-1^ (KBr): 1652 (CO ring, amide).

### Refinement

All the H atoms were positioned geometrically and refined using a riding model: C—H = 0.93 and 0.97 Å for CH and CH_2_ H atoms, respectively, with *U*_iso_(H) = 1.2*U*_eq_(C).

### Crystal data

**Table:**

C_29_H_27_ClN_4_O_2_	*Z* = 2
*M**_r_* = 499.00	*F*(000) = 524
Triclinic, *P*1	*D*_x_ = 1.316 Mg m^−^^3^
Hall symbol: -P 1	Mo *K*α radiation, λ = 0.71073 Å
*a* = 10.7929 (10) Å	Cell parameters from 24474 reflections
*b* = 10.8527 (10) Å	θ = 2.2–27.9°
*c* = 12.7815 (13) Å	µ = 0.19 mm^−^^1^
α = 97.745 (8)°	*T* = 296 K
β = 104.041 (7)°	Prism, colourless
γ = 115.635 (7)°	0.66 × 0.53 × 0.35 mm
*V* = 1259.3 (2) Å^3^	

### Data collection

**Table:**

Stoe IPDS 2 diffractometer	4945 independent reflections
Radiation source: sealed X-ray tube, 12 x 0.4 mm long-fine focus	3837 reflections with *I* > 2σ(*I*)
Plane graphite monochromator	*R*_int_ = 0.059
Detector resolution: 6.67 pixels mm^-1^	θ_max_ = 26.0°, θ_min_ = 2.2°
ω scans	*h* = −13→13
Absorption correction: integration (*X-RED32*; Stoe & Cie, 2002)	*k* = −13→13
*T*_min_ = 0.784, *T*_max_ = 0.948	*l* = −15→15
15922 measured reflections	

### Refinement

**Table:**

Refinement on *F*^2^	Secondary atom site location: difference Fourier map
Least-squares matrix: full	Hydrogen site location: inferred from neighbouring sites
*R*[*F*^2^ > 2σ(*F*^2^)] = 0.041	H-atom parameters constrained
*wR*(*F*^2^) = 0.111	*w* = 1/[σ^2^(*F*_o_^2^) + (0.0478*P*)^2^ + 0.165*P*] where *P* = (*F*_o_^2^ + 2*F*_c_^2^)/3
*S* = 1.05	(Δ/σ)_max_ = 0.001
4945 reflections	Δρ_max_ = 0.29 e Å^−^^3^
326 parameters	Δρ_min_ = −0.37 e Å^−^^3^
0 restraints	Extinction correction: *SHELXL97* (Sheldrick, 2008), FC^*^=KFC[1+0.001XFC^2^Λ^3^/SIN(2Θ)]^-1/4^
Primary atom site location: structure-invariant direct methods	Extinction coefficient: 0.021 (2)

### Special details

**Table:**

Geometry. Bond distances, angles *etc*. have been calculated using the rounded fractional coordinates. All su's are estimated from the variances of the (full) variance-covariance matrix. The cell e.s.d.'s are taken into account in the estimation of distances, angles and torsion angles
Refinement. Refinement on *F*^2^ for ALL reflections except those flagged by the user for potential systematic errors. Weighted *R*-factors *wR* and all goodnesses of fit *S* are based on *F*^2^, conventional *R*-factors *R* are based on *F*, with *F* set to zero for negative *F*^2^. The observed criterion of *F*^2^ > σ(*F*^2^) is used only for calculating -*R*-factor-obs *etc*. and is not relevant to the choice of reflections for refinement. *R*-factors based on *F*^2^ are statistically about twice as large as those based on *F*, and *R*-factors based on ALL data will be even larger.

### Fractional atomic coordinates and isotropic or equivalent isotropic displacement parameters (Å^2^)

**Table:**

	*x*	*y*	*z*	*U*_iso_*/*U*_eq_	
Cl1	1.08842 (7)	1.37656 (7)	0.23195 (6)	0.0985 (3)	
O1	0.77526 (16)	0.94002 (13)	0.89191 (12)	0.0766 (5)	
O2	0.37402 (15)	0.69982 (19)	0.55504 (11)	0.0806 (6)	
N1	0.63418 (14)	0.70263 (13)	0.85637 (11)	0.0502 (4)	
N2	0.61006 (14)	0.57387 (13)	0.87067 (10)	0.0470 (4)	
N3	0.52244 (15)	0.69229 (17)	0.46036 (11)	0.0588 (5)	
N4	0.68833 (14)	0.88867 (16)	0.35873 (11)	0.0550 (5)	
C1	0.75365 (19)	0.83035 (17)	0.91999 (14)	0.0557 (5)	
C2	0.84157 (19)	0.82159 (17)	1.01957 (14)	0.0560 (5)	
C3	0.81424 (16)	0.69621 (16)	1.04284 (12)	0.0475 (5)	
C4	0.69752 (16)	0.56941 (15)	0.95936 (12)	0.0446 (4)	
C5	0.66902 (16)	0.42329 (15)	0.96261 (12)	0.0445 (4)	
C6	0.7836 (2)	0.39377 (19)	0.99172 (15)	0.0587 (6)	
C7	0.7563 (2)	0.2564 (2)	0.98782 (17)	0.0680 (7)	
C8	0.6157 (2)	0.1486 (2)	0.95736 (16)	0.0695 (7)	
C9	0.5011 (2)	0.17680 (18)	0.93023 (15)	0.0612 (6)	
C10	0.52791 (18)	0.31370 (16)	0.93212 (13)	0.0505 (5)	
C11	0.89780 (17)	0.69523 (16)	1.15342 (13)	0.0493 (5)	
C12	1.04711 (19)	0.78512 (19)	1.20005 (16)	0.0609 (6)	
C13	1.1225 (2)	0.7904 (2)	1.30592 (18)	0.0758 (7)	
C14	1.0503 (3)	0.7066 (2)	1.36637 (17)	0.0793 (8)	
C15	0.9027 (2)	0.6189 (2)	1.32177 (15)	0.0679 (7)	
C16	0.8258 (2)	0.61227 (18)	1.21591 (14)	0.0556 (5)	
C17	0.53089 (18)	0.69840 (18)	0.75579 (13)	0.0533 (5)	
C18	0.58384 (18)	0.69042 (19)	0.65707 (13)	0.0546 (5)	
C19	0.48415 (18)	0.69447 (18)	0.55362 (14)	0.0545 (5)	
C20	0.45077 (19)	0.7272 (2)	0.36582 (15)	0.0644 (6)	
C21	0.54978 (19)	0.8760 (2)	0.36406 (15)	0.0622 (6)	
C22	0.76026 (19)	0.8518 (2)	0.45262 (14)	0.0579 (6)	
C23	0.66045 (19)	0.7045 (2)	0.45535 (15)	0.0601 (6)	
C24	0.78162 (17)	1.00788 (18)	0.33070 (13)	0.0526 (5)	
C25	0.7588 (2)	1.1234 (2)	0.32311 (16)	0.0648 (6)	
C26	0.8519 (2)	1.2354 (2)	0.29196 (18)	0.0739 (7)	
C27	0.9681 (2)	1.2340 (2)	0.26832 (16)	0.0665 (6)	
C28	0.9917 (2)	1.1201 (2)	0.27347 (16)	0.0643 (6)	
C29	0.89932 (19)	1.00883 (19)	0.30419 (15)	0.0592 (6)	
H2	0.92010	0.90490	1.06990	0.0670*	
H6	0.87930	0.46690	1.01400	0.0700*	
H7	0.83350	0.23700	1.00590	0.0820*	
H8	0.59780	0.05620	0.95500	0.0830*	
H9	0.40580	0.10380	0.91070	0.0740*	
H10	0.45020	0.33220	0.91270	0.0610*	
H12	1.09660	0.84210	1.15980	0.0730*	
H13	1.22270	0.85080	1.33670	0.0910*	
H14	1.10180	0.70970	1.43740	0.0950*	
H15	0.85390	0.56340	1.36310	0.0810*	
H16	0.72550	0.55220	1.18610	0.0670*	
H17A	0.52040	0.78290	0.76920	0.0640*	
H17B	0.43600	0.61620	0.73890	0.0640*	
H18A	0.68130	0.76950	0.67610	0.0660*	
H18B	0.58920	0.60320	0.64150	0.0660*	
H20A	0.36040	0.72030	0.37220	0.0770*	
H20B	0.42740	0.66010	0.29630	0.0770*	
H21A	0.50310	0.89750	0.29950	0.0750*	
H21B	0.56740	0.94370	0.43110	0.0750*	
H22A	0.78630	0.91950	0.52250	0.0700*	
H22B	0.84920	0.85650	0.44460	0.0700*	
H23A	0.64250	0.63590	0.38880	0.0720*	
H23B	0.70680	0.68350	0.52030	0.0720*	
H25	0.68000	1.12540	0.33910	0.0780*	
H26	0.83530	1.31200	0.28710	0.0890*	
H28	1.06990	1.11840	0.25620	0.0770*	
H29	0.91600	0.93200	0.30730	0.0710*	

### Atomic displacement parameters (Å^2^)

**Table:**

	*U*^11^	*U*^22^	*U*^33^	*U*^12^	*U*^13^	*U*^23^
Cl1	0.0811 (4)	0.0793 (4)	0.1288 (5)	0.0272 (3)	0.0379 (4)	0.0461 (4)
O1	0.0907 (10)	0.0489 (7)	0.0827 (9)	0.0260 (7)	0.0238 (7)	0.0331 (6)
O2	0.0747 (9)	0.1370 (13)	0.0629 (8)	0.0719 (10)	0.0320 (7)	0.0370 (8)
N1	0.0563 (8)	0.0463 (7)	0.0512 (7)	0.0236 (6)	0.0212 (6)	0.0214 (6)
N2	0.0524 (7)	0.0427 (6)	0.0488 (7)	0.0214 (6)	0.0215 (6)	0.0184 (5)
N3	0.0588 (8)	0.0806 (10)	0.0523 (8)	0.0420 (8)	0.0234 (6)	0.0261 (7)
N4	0.0508 (8)	0.0698 (9)	0.0524 (8)	0.0349 (7)	0.0176 (6)	0.0207 (7)
C1	0.0640 (10)	0.0456 (8)	0.0587 (9)	0.0226 (8)	0.0263 (8)	0.0215 (7)
C2	0.0590 (10)	0.0425 (8)	0.0540 (9)	0.0145 (7)	0.0175 (7)	0.0141 (7)
C3	0.0484 (8)	0.0461 (8)	0.0487 (8)	0.0202 (7)	0.0213 (7)	0.0159 (6)
C4	0.0464 (8)	0.0442 (7)	0.0463 (8)	0.0202 (7)	0.0215 (6)	0.0167 (6)
C5	0.0521 (8)	0.0431 (7)	0.0421 (7)	0.0234 (7)	0.0198 (6)	0.0147 (6)
C6	0.0562 (10)	0.0582 (10)	0.0687 (10)	0.0310 (8)	0.0251 (8)	0.0194 (8)
C7	0.0813 (13)	0.0719 (12)	0.0746 (12)	0.0530 (11)	0.0305 (10)	0.0278 (10)
C8	0.1002 (15)	0.0526 (10)	0.0695 (11)	0.0433 (11)	0.0336 (11)	0.0268 (9)
C9	0.0687 (11)	0.0464 (9)	0.0605 (10)	0.0202 (8)	0.0213 (8)	0.0184 (7)
C10	0.0539 (9)	0.0462 (8)	0.0501 (8)	0.0224 (7)	0.0174 (7)	0.0160 (7)
C11	0.0519 (9)	0.0457 (8)	0.0483 (8)	0.0233 (7)	0.0159 (7)	0.0099 (6)
C12	0.0536 (10)	0.0559 (10)	0.0696 (11)	0.0258 (8)	0.0190 (8)	0.0125 (8)
C13	0.0593 (11)	0.0720 (12)	0.0794 (13)	0.0338 (10)	0.0009 (10)	0.0048 (10)
C14	0.0937 (16)	0.0884 (14)	0.0572 (11)	0.0577 (13)	0.0049 (10)	0.0136 (10)
C15	0.0880 (14)	0.0730 (12)	0.0547 (10)	0.0479 (11)	0.0235 (9)	0.0231 (9)
C16	0.0602 (10)	0.0568 (9)	0.0512 (9)	0.0284 (8)	0.0190 (7)	0.0180 (7)
C17	0.0561 (9)	0.0576 (9)	0.0544 (9)	0.0297 (8)	0.0225 (7)	0.0250 (7)
C18	0.0559 (9)	0.0624 (10)	0.0555 (9)	0.0327 (8)	0.0225 (7)	0.0248 (8)
C19	0.0556 (9)	0.0622 (10)	0.0532 (9)	0.0328 (8)	0.0211 (7)	0.0179 (7)
C20	0.0545 (10)	0.0927 (13)	0.0500 (9)	0.0378 (10)	0.0173 (7)	0.0246 (9)
C21	0.0595 (10)	0.0881 (13)	0.0544 (9)	0.0461 (10)	0.0196 (8)	0.0281 (9)
C22	0.0567 (10)	0.0717 (11)	0.0546 (9)	0.0396 (9)	0.0170 (7)	0.0182 (8)
C23	0.0649 (10)	0.0738 (11)	0.0610 (10)	0.0439 (10)	0.0297 (8)	0.0249 (9)
C24	0.0527 (9)	0.0586 (9)	0.0440 (8)	0.0291 (8)	0.0111 (7)	0.0084 (7)
C25	0.0676 (11)	0.0726 (11)	0.0692 (11)	0.0439 (10)	0.0275 (9)	0.0215 (9)
C26	0.0813 (13)	0.0645 (11)	0.0876 (14)	0.0436 (11)	0.0283 (11)	0.0267 (10)
C27	0.0609 (11)	0.0609 (10)	0.0667 (11)	0.0238 (9)	0.0152 (9)	0.0165 (8)
C28	0.0529 (10)	0.0674 (11)	0.0674 (11)	0.0273 (9)	0.0181 (8)	0.0129 (9)
C29	0.0568 (10)	0.0604 (10)	0.0636 (10)	0.0326 (9)	0.0191 (8)	0.0136 (8)

### Geometric parameters (Å, º)

**Table:**

Cl1—C27	1.745 (2)	C24—C25	1.389 (3)
O1—C1	1.231 (2)	C24—C29	1.388 (3)
O2—C19	1.219 (3)	C25—C26	1.383 (3)
N1—N2	1.3532 (19)	C26—C27	1.365 (3)
N1—C1	1.373 (2)	C27—C28	1.372 (3)
N1—C17	1.464 (2)	C28—C29	1.372 (3)
N2—C4	1.309 (2)	C2—H2	0.9300
N3—C19	1.354 (2)	C6—H6	0.9300
N3—C20	1.458 (2)	C7—H7	0.9300
N3—C23	1.455 (3)	C8—H8	0.9300
N4—C21	1.461 (3)	C9—H9	0.9300
N4—C22	1.466 (2)	C10—H10	0.9300
N4—C24	1.410 (2)	C12—H12	0.9300
C1—C2	1.433 (3)	C13—H13	0.9300
C2—C3	1.353 (2)	C14—H14	0.9300
C3—C4	1.439 (2)	C15—H15	0.9300
C3—C11	1.485 (2)	C16—H16	0.9300
C4—C5	1.488 (2)	C17—H17A	0.9700
C5—C6	1.386 (3)	C17—H17B	0.9700
C5—C10	1.381 (3)	C18—H18A	0.9700
C6—C7	1.380 (3)	C18—H18B	0.9700
C7—C8	1.370 (3)	C20—H20A	0.9700
C8—C9	1.375 (3)	C20—H20B	0.9700
C9—C10	1.381 (3)	C21—H21A	0.9700
C11—C12	1.386 (3)	C21—H21B	0.9700
C11—C16	1.394 (3)	C22—H22A	0.9700
C12—C13	1.379 (3)	C22—H22B	0.9700
C13—C14	1.378 (3)	C23—H23A	0.9700
C14—C15	1.367 (4)	C23—H23B	0.9700
C15—C16	1.379 (3)	C25—H25	0.9300
C17—C18	1.513 (3)	C26—H26	0.9300
C18—C19	1.508 (3)	C28—H28	0.9300
C20—C21	1.510 (3)	C29—H29	0.9300
C22—C23	1.504 (3)		
			
Cl1···H7^i^	3.0900	H6···C11	2.7500
O1···C18	3.252 (2)	H6···H6^vii^	2.5200
O1···C12^ii^	3.323 (2)	H7···Cl1^xi^	3.0900
O2···C25^iii^	3.289 (3)	H7···H12^vii^	2.5800
O1···H17A	2.4600	H8···O1^xiv^	2.9100
O1···H8^iv^	2.9100	H8···C8^viii^	3.0000
O1···H2^ii^	2.8400	H8···C9^viii^	3.0200
O1···H12^ii^	2.4400	H8···H8^viii^	2.5700
O1···H18A	2.7800	H8···H9^viii^	2.6000
O2···H17B	2.6700	H9···C24^v^	2.9900
O2···H20A	2.3500	H9···H8^viii^	2.6000
O2···H17A	2.6100	H10···N2	2.6600
O2···H25^iii^	2.5300	H10···C3^vi^	2.9400
N3···N4	2.830 (2)	H10···C4^vi^	2.9400
N4···N3	2.830 (2)	H12···C2	2.7900
N2···H10	2.6600	H12···H2	2.3800
N2···H20B^v^	2.9000	H12···O1^ii^	2.4400
N3···H18B^v^	2.8600	H12···C7^vii^	3.1000
C5···C16	3.178 (2)	H12···H7^vii^	2.5800
C6···C11	3.154 (3)	H13···H20A^x^	2.4700
C6···C16	3.262 (3)	H14···C25^ii^	2.9700
C9···C21^v^	3.595 (3)	H16···C4	2.8800
C9···C24^v^	3.524 (3)	H16···C5	2.8000
C11···C6	3.154 (3)	H16···H17B^vi^	2.4100
C12···O1^ii^	3.323 (2)	H17A···O1	2.4600
C16···C5	3.178 (2)	H17A···O2	2.6100
C16···C6	3.262 (3)	H17B···O2	2.6700
C18···O1	3.252 (2)	H17B···C16^vi^	3.1000
C21···C9^v^	3.595 (3)	H17B···H16^vi^	2.4100
C24···C9^v^	3.524 (3)	H18A···O1	2.7800
C25···O2^iii^	3.289 (3)	H18A···C1	2.9300
C1···H18A	2.9300	H18A···C23	2.7400
C2···H12	2.7900	H18A···H23B	2.2100
C3···H6	2.8700	H18A···C28^xiii^	3.0600
C3···H10^vi^	2.9400	H18A···H28^xiii^	2.2800
C4···H16	2.8800	H18B···C23	2.8800
C4···H10^vi^	2.9400	H18B···H23B	2.2700
C5···H20B^v^	3.0900	H18B···N3^v^	2.8600
C5···H16	2.8000	H18B···C23^v^	3.0700
C7···H12^vii^	3.1000	H20A···O2	2.3500
C8···H8^viii^	3.0000	H20A···C13^xv^	2.9500
C9···H21A^v^	2.9200	H20A···H13^xv^	2.4700
C9···H8^viii^	3.0200	H20B···H23A	2.4800
C11···H29^ix^	2.9200	H20B···N2^v^	2.9000
C11···H6	2.7500	H20B···C5^v^	3.0900
C12···H2	2.7100	H21A···C25	2.6900
C12···H29^ix^	2.9500	H21A···H25	2.2600
C13···H20A^x^	2.9500	H21A···C9^v^	2.9200
C15···H26^xi^	3.0300	H21B···C25	2.9300
C16···H17B^vi^	3.1000	H21B···H22A	2.5100
C18···H23B	2.4500	H21B···H25	2.4500
C21···H25	2.5600	H22A···H21B	2.5100
C22···H29	2.7800	H22B···C29	2.6000
C23···H18B	2.8800	H22B···H29	2.1800
C23···H18B^v^	3.0700	H23A···H20B	2.4800
C23···H18A	2.7400	H23B···C18	2.4500
C24···H9^v^	2.9900	H23B···H18A	2.2100
C25···H21A	2.6900	H23B···H18B	2.2700
C25···H21B	2.9300	H25···C21	2.5600
C25···H14^ii^	2.9700	H25···H21A	2.2600
C28···H2^xii^	2.9400	H25···H21B	2.4500
C28···H18A^xiii^	3.0600	H25···O2^iii^	2.5300
C29···H22B	2.6000	H26···C15^i^	3.0300
H2···C12	2.7100	H28···H18A^xiii^	2.2800
H2···C28^ix^	2.9400	H29···C11^xii^	2.9200
H2···H12	2.3800	H29···C12^xii^	2.9500
H2···O1^ii^	2.8400	H29···C22	2.7800
H6···C3	2.8700	H29···H22B	2.1800
			
N2—N1—C1	125.35 (15)	C7—C6—H6	120.00
N2—N1—C17	114.86 (13)	C6—C7—H7	120.00
C1—N1—C17	119.35 (14)	C8—C7—H7	120.00
N1—N2—C4	118.26 (13)	C7—C8—H8	120.00
C19—N3—C20	120.13 (18)	C9—C8—H8	120.00
C19—N3—C23	125.31 (16)	C8—C9—H9	120.00
C20—N3—C23	111.42 (15)	C10—C9—H9	120.00
C21—N4—C22	111.60 (15)	C5—C10—H10	120.00
C21—N4—C24	118.29 (16)	C9—C10—H10	120.00
C22—N4—C24	115.21 (16)	C11—C12—H12	120.00
O1—C1—N1	120.49 (17)	C13—C12—H12	120.00
O1—C1—C2	125.55 (17)	C12—C13—H13	120.00
N1—C1—C2	113.92 (15)	C14—C13—H13	120.00
C1—C2—C3	122.23 (16)	C13—C14—H14	120.00
C2—C3—C4	116.98 (15)	C15—C14—H14	120.00
C2—C3—C11	119.65 (15)	C14—C15—H15	120.00
C4—C3—C11	123.27 (14)	C16—C15—H15	120.00
N2—C4—C3	122.15 (14)	C11—C16—H16	120.00
N2—C4—C5	114.01 (13)	C15—C16—H16	120.00
C3—C4—C5	123.79 (14)	N1—C17—H17A	110.00
C4—C5—C6	120.46 (16)	N1—C17—H17B	110.00
C4—C5—C10	120.58 (17)	C18—C17—H17A	110.00
C6—C5—C10	118.90 (16)	C18—C17—H17B	110.00
C5—C6—C7	120.31 (19)	H17A—C17—H17B	108.00
C6—C7—C8	120.2 (2)	C17—C18—H18A	109.00
C7—C8—C9	120.12 (19)	C17—C18—H18B	109.00
C8—C9—C10	119.85 (19)	C19—C18—H18A	109.00
C5—C10—C9	120.63 (19)	C19—C18—H18B	109.00
C3—C11—C12	120.49 (16)	H18A—C18—H18B	108.00
C3—C11—C16	120.48 (17)	N3—C20—H20A	110.00
C12—C11—C16	118.84 (16)	N3—C20—H20B	110.00
C11—C12—C13	120.20 (18)	C21—C20—H20A	110.00
C12—C13—C14	120.4 (2)	C21—C20—H20B	110.00
C13—C14—C15	119.9 (2)	H20A—C20—H20B	108.00
C14—C15—C16	120.38 (19)	N4—C21—H21A	110.00
C11—C16—C15	120.28 (19)	N4—C21—H21B	110.00
N1—C17—C18	110.42 (17)	C20—C21—H21A	110.00
C17—C18—C19	111.72 (17)	C20—C21—H21B	110.00
O2—C19—N3	121.94 (17)	H21A—C21—H21B	108.00
O2—C19—C18	120.78 (17)	N4—C22—H22A	110.00
N3—C19—C18	117.29 (18)	N4—C22—H22B	110.00
N3—C20—C21	109.95 (16)	C23—C22—H22A	110.00
N4—C21—C20	110.00 (17)	C23—C22—H22B	110.00
N4—C22—C23	110.26 (16)	H22A—C22—H22B	108.00
N3—C23—C22	110.62 (18)	N3—C23—H23A	110.00
N4—C24—C25	123.46 (19)	N3—C23—H23B	110.00
N4—C24—C29	119.06 (17)	C22—C23—H23A	110.00
C25—C24—C29	117.41 (18)	C22—C23—H23B	110.00
C24—C25—C26	120.8 (2)	H23A—C23—H23B	108.00
C25—C26—C27	120.3 (2)	C24—C25—H25	120.00
Cl1—C27—C26	120.62 (17)	C26—C25—H25	120.00
Cl1—C27—C28	119.29 (18)	C25—C26—H26	120.00
C26—C27—C28	120.1 (2)	C27—C26—H26	120.00
C27—C28—C29	119.7 (2)	C27—C28—H28	120.00
C24—C29—C28	121.76 (19)	C29—C28—H28	120.00
C1—C2—H2	119.00	C24—C29—H29	119.00
C3—C2—H2	119.00	C28—C29—H29	119.00
C5—C6—H6	120.00		
			
C1—N1—N2—C4	8.3 (3)	C2—C3—C4—N2	−7.7 (3)
C17—N1—N2—C4	−179.47 (16)	N2—C4—C5—C10	−42.1 (2)
N2—N1—C1—O1	170.70 (18)	C3—C4—C5—C6	−42.2 (2)
C17—N1—C1—O1	−1.2 (3)	C3—C4—C5—C10	140.53 (18)
N2—N1—C1—C2	−11.6 (3)	N2—C4—C5—C6	135.15 (17)
C17—N1—C1—C2	176.54 (17)	C6—C5—C10—C9	−0.2 (2)
N2—N1—C17—C18	−88.78 (18)	C10—C5—C6—C7	1.4 (3)
C1—N1—C17—C18	84.0 (2)	C4—C5—C10—C9	177.05 (15)
N1—N2—C4—C3	2.0 (3)	C4—C5—C6—C7	−175.90 (16)
N1—N2—C4—C5	−175.41 (15)	C5—C6—C7—C8	−1.4 (3)
C20—N3—C19—O2	13.4 (3)	C6—C7—C8—C9	0.2 (3)
C23—N3—C19—O2	171.58 (19)	C7—C8—C9—C10	1.0 (3)
C23—N3—C20—C21	−58.0 (2)	C8—C9—C10—C5	−1.0 (3)
C19—N3—C20—C21	103.0 (2)	C12—C11—C16—C15	−0.7 (3)
C23—N3—C19—C18	−8.3 (3)	C3—C11—C16—C15	−175.65 (18)
C19—N3—C23—C22	−102.3 (2)	C16—C11—C12—C13	0.8 (3)
C20—N3—C19—C18	−166.45 (16)	C3—C11—C12—C13	175.75 (18)
C20—N3—C23—C22	57.50 (19)	C11—C12—C13—C14	0.0 (3)
C24—N4—C21—C20	165.70 (14)	C12—C13—C14—C15	−0.8 (4)
C21—N4—C22—C23	56.4 (2)	C13—C14—C15—C16	0.9 (4)
C22—N4—C21—C20	−57.14 (19)	C14—C15—C16—C11	−0.2 (3)
C24—N4—C22—C23	−165.05 (16)	N1—C17—C18—C19	−176.70 (14)
C22—N4—C24—C25	−126.42 (19)	C17—C18—C19—N3	178.08 (16)
C21—N4—C24—C29	−167.77 (15)	C17—C18—C19—O2	−1.8 (3)
C21—N4—C24—C25	9.3 (2)	N3—C20—C21—N4	57.2 (2)
C22—N4—C24—C29	56.6 (2)	N4—C22—C23—N3	−55.78 (19)
N1—C1—C2—C3	5.0 (3)	N4—C24—C25—C26	−178.06 (17)
O1—C1—C2—C3	−177.4 (2)	C29—C24—C25—C26	−1.0 (3)
C1—C2—C3—C11	−172.67 (19)	N4—C24—C29—C28	178.30 (16)
C1—C2—C3—C4	3.8 (3)	C25—C24—C29—C28	1.1 (3)
C2—C3—C4—C5	169.46 (18)	C24—C25—C26—C27	−0.1 (3)
C11—C3—C4—C5	−14.2 (3)	C25—C26—C27—Cl1	−178.59 (16)
C4—C3—C11—C12	140.70 (19)	C25—C26—C27—C28	1.1 (3)
C4—C3—C11—C16	−44.4 (3)	Cl1—C27—C28—C29	178.70 (15)
C11—C3—C4—N2	168.63 (18)	C26—C27—C28—C29	−1.0 (3)
C2—C3—C11—C12	−43.0 (3)	C27—C28—C29—C24	−0.1 (3)
C2—C3—C11—C16	131.8 (2)		

Symmetry codes: (i) *x*, *y*+1, *z*−1; (ii) −*x*+2, −*y*+2, −*z*+2; (iii) −*x*+1, −*y*+2, −*z*+1; (iv) *x*, *y*+1, *z*; (v) −*x*+1, −*y*+1, −*z*+1; (vi) −*x*+1, −*y*+1, −*z*+2; (vii) −*x*+2, −*y*+1, −*z*+2; (viii) −*x*+1, −*y*, −*z*+2; (ix) *x*, *y*, *z*+1; (x) *x*+1, *y*, *z*+1; (xi) *x*, *y*−1, *z*+1; (xii) *x*, *y*, *z*−1; (xiii) −*x*+2, −*y*+2, −*z*+1; (xiv) *x*, *y*−1, *z*; (xv) *x*−1, *y*, *z*−1.

### Hydrogen-bond geometry (Å, º)

Cg1 and Cg4 are the centroids of the N1/N2/C1–C4 and C11–C16 rings, respectively.

**Table:**

*D*—H···*A*	*D*—H	H···*A*	*D*···*A*	*D*—H···*A*
C12—H12···O1^ii^	0.93	2.44	3.323 (2)	158
C25—H25···O2^iii^	0.93	2.53	3.289 (3)	139
C10—H10···*Cg*1^vi^	0.93	2.88	3.431 (2)	119
C29—H29···*Cg*4^xii^	0.93	2.86	3.762 (2)	165

Symmetry codes: (ii) −*x*+2, −*y*+2, −*z*+2; (iii) −*x*+1, −*y*+2, −*z*+1; (vi) −*x*+1, −*y*+1, −*z*+2; (xii) *x*, *y*, *z*−1.
